# Actinomycosis Associated With a Nasal Rhinolith

**DOI:** 10.7759/cureus.107742

**Published:** 2026-04-26

**Authors:** Maryam Qader, Shooq Abdulla, Zainab Harb, Kawthar Qader

**Affiliations:** 1 Otolaryngology - Head and Neck Surgery, Salmaniya Medical Complex, Manama, BHR; 2 Histopathology, Salmaniya Medical Complex, Manama, BHR

**Keywords:** actinomycosis, epistaxis, nose, rhinolith, sinonasal

## Abstract

Rhinolithiasis is a rare entity characterised by the formation of calcified masses within the nasal cavity, resulting in chronic unilateral symptoms. We report a case of a patient presenting with a two-year history of unilateral epistaxis and foul nasal discharge. Clinical examination and computed tomography revealed a calcified mass in the right nasal cavity, which was endoscopically removed. Histopathological examination of the mass revealed *Actinomyces* infection. The patient was successfully managed with a three-month course of oral amoxicillin. This case highlights the importance of histopathological evaluation for rhinoliths, as they can act as an anaerobic site for opportunistic infections such as actinomycosis.

## Introduction

Rhinolithiasis is an uncommon condition characterised by the formation of calcified masses (rhinoliths) within the nasal cavity. These structures usually develop gradually over several years through the deposition of calcium and magnesium salts around a central nidus [1]. Although patients may initially be asymptomatic, the condition can eventually present with chronic unilateral nasal symptoms, such as nasal obstruction and purulent rhinorrhea [1].

Species of *Actinomyces*, which are anaerobic bacteria commonly found in the normal flora of the oropharynx, can cause opportunistic infections known as actinomycosis when mucosal barriers are disrupted [2,3]. These infections typically manifest with chronic inflammation, abscess formation, and draining sinus tracts [4]. The presence of a rhinolith may create a favourable anaerobic environment that promotes colonisation and growth of *Actinomyces* [5].

This case report exhibits the rare presentation of an actinomycosis infection associated with a rhinolith and highlights the recognition of such an entity to prevent treatment failure and disease recurrence.

## Case presentation

A 38-year-old female with a known history of medically managed hypothyroidism presented to our otorhinolaryngology clinic with a two-year history of recurrent unilateral right-sided epistaxis and foul-smelling nasal discharge. She explained that the odour was quite noticeable by herself and by others as well, and that this had been causing her significant embarrassment. She denied experiencing any nasal blockage, hyposmia, or any other symptoms. She did not undergo any previous nasal surgeries, and her social and family history were unremarkable.

**Figure 1:**
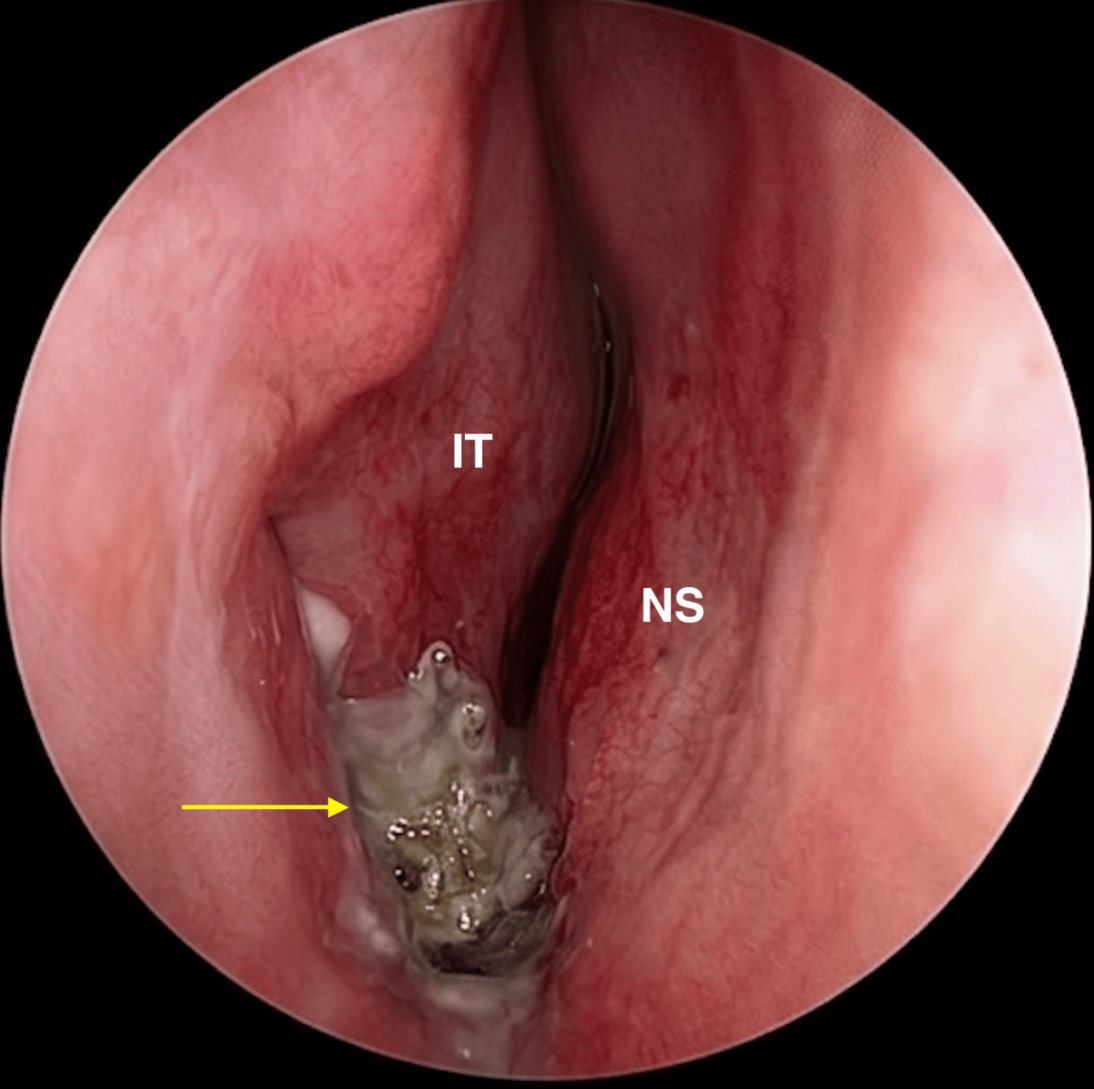
Endoscopic view of the right nasal cavity demonstrating a calcified rhinolith (arrow) along the nasal floor between the inferior turbinate (IT) and nasal septum (NS).

Upon rigid nasal endoscopy, a hard grey matter was visualised in the right nasal cavity, located along the nasal floor, inferior to the inferior turbinate (Figure 1). It was associated with a pronounced and noticeable foul odour and surrounding discharge. This presentation raised the suspicion of a rhinolith. An attempt was made to remove the mass in the outpatient clinic, but the patient could not tolerate the procedure.

**Figure 2:**
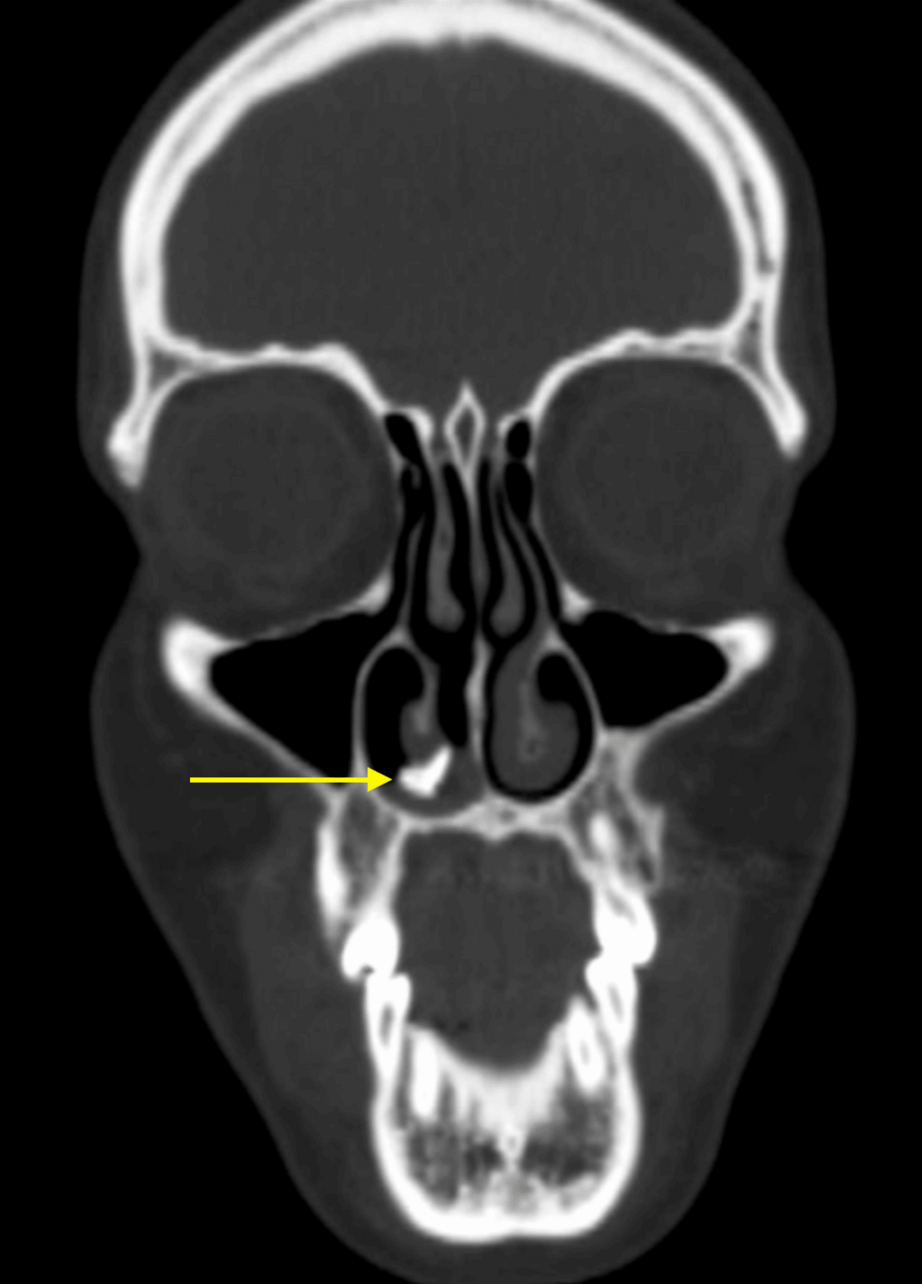
Non-contrast CT scan of the paranasal sinuses demonstrating a well-defined hyperdense calcified lesion along the floor of the right nasal cavity (arrow), consistent with a rhinolith.

Following the examination, a non-contrast computed tomography (CT) scan of the paranasal sinuses was performed. The CT scan revealed hyperdense calcification-like material with surrounding opacification located along the floor of the right nasal cavity just below the inferior turbinate (Figure 2).

**Figure 3:**
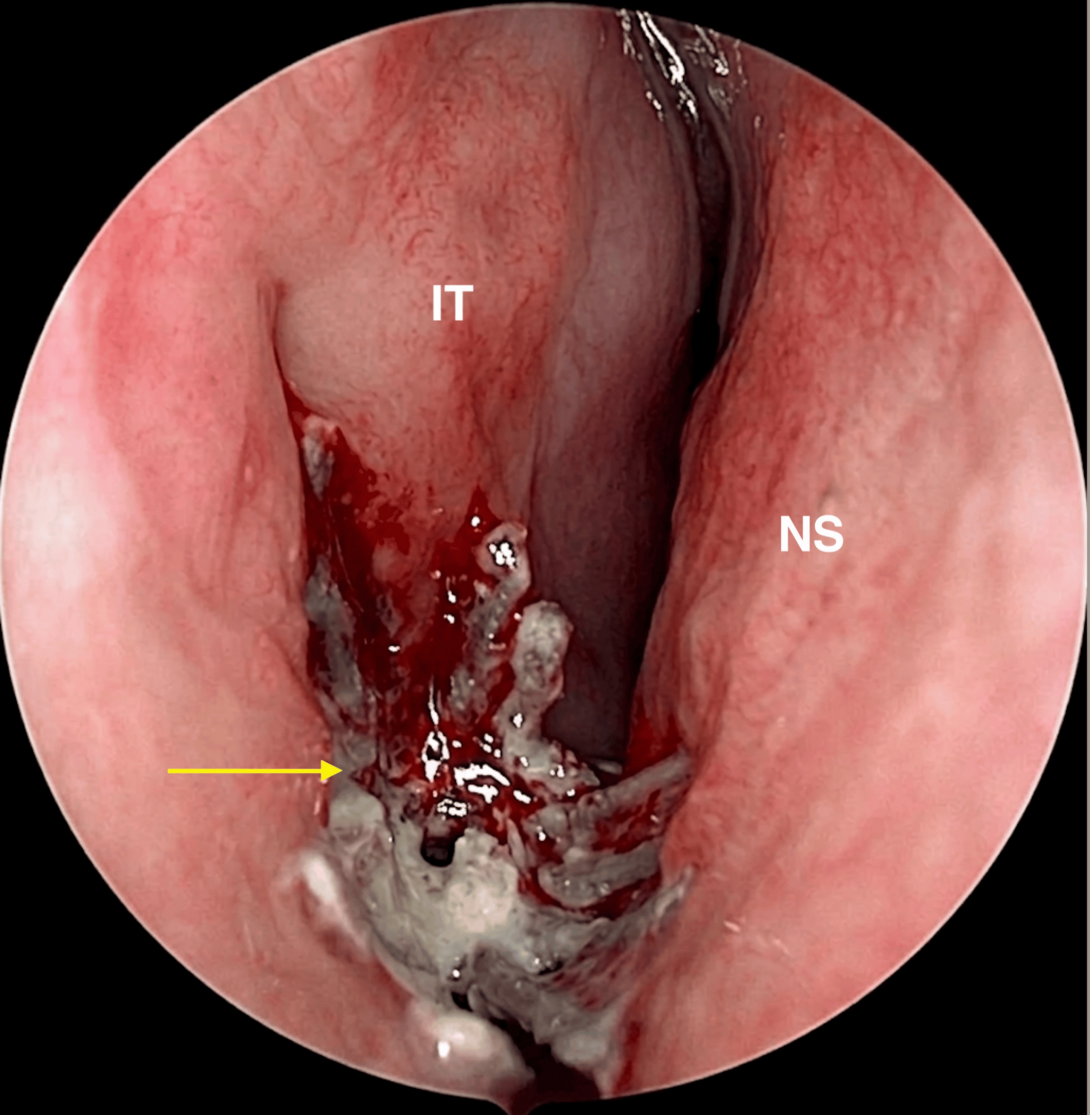
Intraoperative nasal endoscopy demonstrating a rhinolith (arrow) associated with a retained foreign body, with surrounding purulent discharge and crusting. IT: inferior turbinate; NS: nasal septum

**Figure 4:**
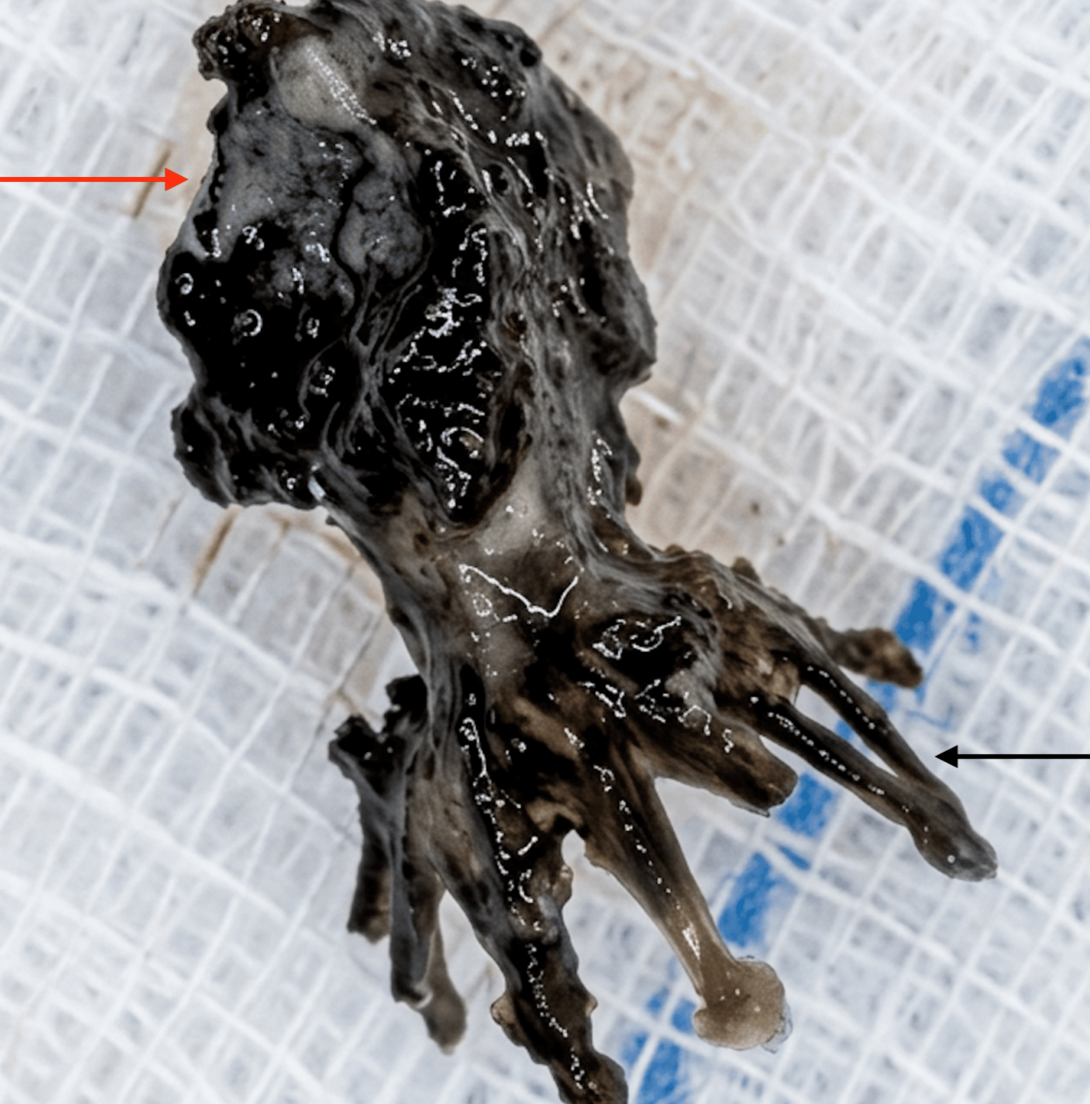
Gross specimen from the right nasal cavity showing a calcified rhinolith (red arrow) encasing a retained foreign body (black arrow).

The patient was therefore scheduled for an endoscopic removal of the mass under general anaesthesia. Intraoperatively, we found a foreign body that was surrounded by green discharge and significant black crusting, which was consistent with a rhinolith (Figure 3). It was wedged into the right inferior meatus and was removed in one piece (Figure 4).

**Figure 5:**
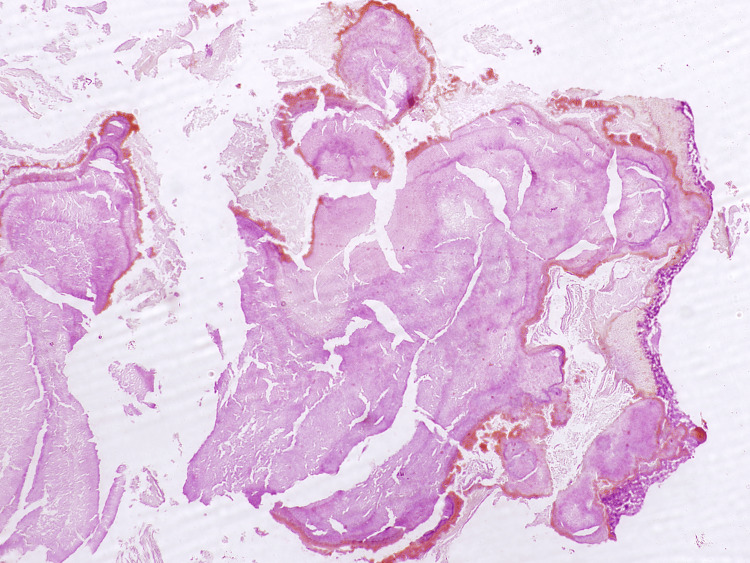
H&E-stained section of a rhinolith demonstrating basophilic filamentous colonies consistent with actinomycosis. 10x magnification

**Figure 6:**
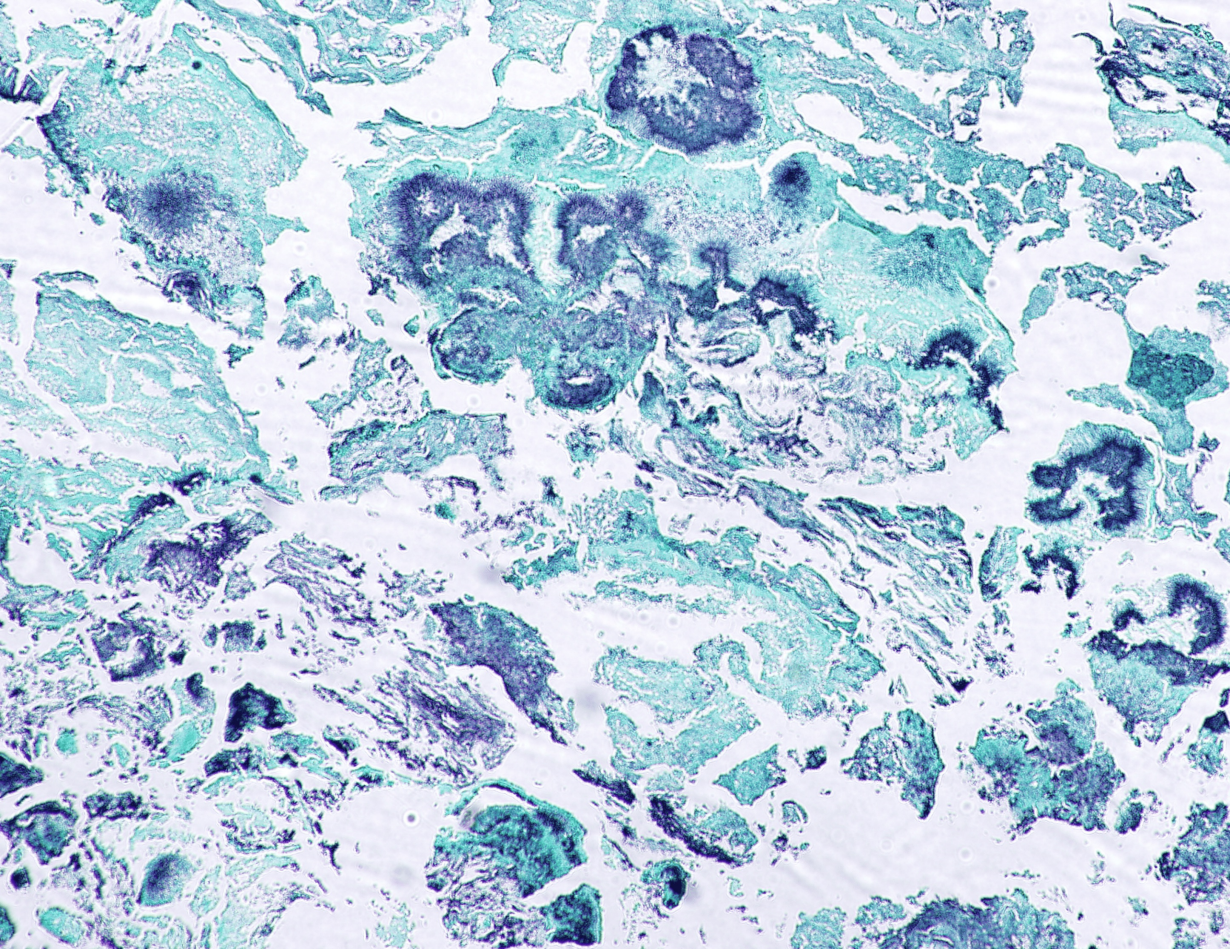
Grocott's methenamine silver (GMS) stain highlighting filamentous organisms within the rhinolith, consistent with actinomycosis. 10x magnification

Histopathological examination of the excised tissue sections revealed calcified fragments composed of basophilic, filamentous bacterial organisms exhibiting a characteristic "starburst" appearance (Figure 5). These structures were consistent with Actinomycotic sulfur granules. The organisms stained positive with Grocott's methenamine silver (GMS) stain, further confirming the presence of *Actinomyces* species (Figure 6).

Following confirmation of actinomycosis in the rhinolith, the Infectious Disease Department was consulted, and the patient was started on a course of oral antibiotic therapy, consisting of amoxicillin 1 g taken three times daily. The treatment continued for a duration of three months.

Postoperatively, within the first week, the patient reported complete resolution of the foul-smelling nasal discharge and epistaxis. She was followed closely in the outpatient clinics throughout the duration of the oral treatment and was monitored for possible recurrence or residual disease.

Endoscopic nasal examination at two weeks, three months, and six months was performed and confirmed healthy and healed nasal mucosa with no evidence of disease recurrence. On the last visit six months after the surgery, she reported that she has since returned to her normal daily activity with a significantly improved quality of life and that she no longer felt embarrassed in social settings.

## Discussion

Rhinolithiasis complicated by actinomycosis is a rare clinical finding. *Actinomyces* species are filamentous Gram-positive bacilli that are primarily part of the normal microbial flora of the oropharynx, gastrointestinal tract, and urogenital tract [2,4]. The pathogenesis of actinomycosis requires a disruption of normal healthy mucosa and an anaerobic environment created by synergistic bacteria [2,3]. The presence of a rhinolith can lead to a breach of the healthy mucosa through chronic irritation and stasis of secretion, together creating a hypoxic environment suited for the growth of opportunistic anaerobic organisms, such as *Actinomyces *[5]. In our patient, the rhinolith served as the trigger, allowing the opportunistic *Actinomyces* bacteria to become a localized infection, presenting as chronic nasal blockage, epistaxis, and a foul smell from the nasal cavity.

The diagnosis of actinomycosis in the sinonasal cavity is challenging due to its rarity and being misdiagnosed as more common sinonasal pathologies, such as chronic rhinosinusitis, fungal balls, or simple foreign bodies [6]. Radiological imaging, particularly CT, is helpful in ruling out other pathologies, identifying the calcified mass, and assessing the extent of the disease, though it cannot definitively diagnose actinomycosis [5]. As seen in our case, the diagnosis heavily relies on histopathological examination of the excised specimen. The hallmark histological finding is the presence of "sulfur granules" that are basophilic masses with radiating eosinophilic filaments that appear as a characteristic "starburst" appearance [2]. The diagnosis can be further confirmed using GMS staining to demonstrate the Gram-positive filamentous branching bacteria at the periphery of the grains [4].

A review of the literature reveals only a handful of similar cases, highlighting the rarity of this dual pathology. Table 1 summarises recent published cases of rhinolithiasis associated with actinomycosis.

**Table 1:** Summary of recent published cases of rhinolithiasis with actinomycosis and comparison with the current case.

Author (Year)	Age/Sex	Clinical Presentation	Treatment Regimen	Outcome
Zalagh et al. (2012) [7]	25/M	Unilateral nasal obstruction and purulent rhinorrhea	Endoscopic removal + Ciprofloxacin (6 weeks)	Complete resolution
Batzakakis et al. (2013) [6]	34/F	Halitosis and unilateral offensive rhinorrhea	Removed through combined Endoscopic and Transoral approach + IV Penicillin (3 days) then Oral Penicillin (4 weeks)	Complete resolution by 6 weeks
Kim et al. (2018) [5]	33/F	Unilateral nasal obstruction, epistaxis, and rhinorrhea	Endoscopic removal + Cefdinir + Mupirocin Nasal irrigation	No recurrence at 6 months
Kamogashira et al. (2020) [8]	39/M	Epistaxis and unilateral cheek pain	Endoscopic removal + IV Penicillin G (1 week) then Oral Amoxicillin (6 months)	No recurrence at 1 year
Harmon et al. (2023) [3]	54/M	Chronic cough, throat pain	Endoscopic + Oral Amoxicillin (4 months)	No recurrence at 5 months
Current Case	38/F	Unilateral epistaxis and foul-smelling nasal discharge	Endoscopic removal + Oral Amoxicillin (3 months)	Complete resolution at 6 months

A review of these published cases reveals a number of notable patterns. The majority of patients presented with chronic unilateral nasal symptoms, most commonly obstruction and rhinorrhea. Treatment of all cases involved surgical removal of the mass followed by antimicrobial therapy. However, the antibiotic choice and duration varied, ranging from six weeks of ciprofloxacin [7] to a combination of intravenous, followed by oral penicillin lasting for six months [8]. A 6-12 month course of antibiotic therapy has been traditionally recommended to prevent relapse in cases of actinomycosis. However, recent evidence suggests that immunocompetent patients who have had complete surgical resection can be managed with shorter courses of antimicrobial therapy, especially in cases of orocervicofacial involvement [2,4]. Our patient achieved complete clinical and radiological resolution with a three-month course of oral amoxicillin after surgical removal. Notably, no recurrence was observed in any of the cases where complete surgical excision was performed, further supporting the crucial role of intraoperative debridement.

## Conclusions

This case underscores several critical points of interest for the practicing clinician. First, it highlights the absolute necessity of submitting all surgically excised nasal masses, including presumed simple rhinoliths, for routine histopathological evaluation to rule out underlying opportunistic infections. Second, it supports the emerging paradigm that a shortened course of targeted oral antibiotics may be sufficient for localised nasal actinomycosis when combined with complete surgical clearance. While our conclusions are drawn from a single case and supported by a limited number of similar reports, they suggest that a high index of suspicion and a tailored combined medical-surgical approach yield excellent patient outcomes.
